# Modulation of Steroidogenic Pathway in Rat Granulosa Cells with Subclinical Cd Exposure and Insulin Resistance: An Impact on Female Fertility

**DOI:** 10.1155/2014/460251

**Published:** 2014-08-19

**Authors:** Muskaan Belani, Nupur Purohit, Prakash Pillai, Sharad Gupta, Sarita Gupta

**Affiliations:** ^1^Department of Biochemistry, Faculty of Science, The M. S. University of Baroda, Vadodara, Gujarat 390 002, India; ^2^Department of Zoology, Faculty of Science, The M. S. University of Baroda, Vadodara, Gujarat 390 002, India; ^3^Gupta's Pathology Laboratory, Vadodara, Gujarat 390 002, India

## Abstract

Changes in lifestyle lead to insulin resistance (IR) in females ultimately predisposing them towards infertility. In addition, cadmium (Cd), an environmental endocrine disruptor, is reported for detrimental effects on granulosa cells, thus leading to ovarian dysfunction. A combination of these factors, lifestyle and environment, seems to play a role in etiology of idiopathic infertility that accounts for 50% amongst the total infertility cases. To address this issue, we made an attempt to investigate the extent of Cd impact on insulin-resistant (IR) granulosa cells. We exposed adult female *Charles Foster* rats to dexamethasone and confirmed IR condition by fasting insulin resistance index (FIRI). On treatment of IR rats with Cd, the preliminary studies demonstrated prolonged estrous cyclicity, decrease in serum estradiol concentrations, abnormal histology of ovary, and increased granulosa cell death. Further gene and protein expression studies of steroidogenic acute regulatory (StAR) protein, 17*β*-hydroxysteroid dehydrogenase (17*β*-HSD), and cytochrome P450 aromatase (CYP19A1) were performed. Protein expression studies demonstrated significant decrease in treated groups when compared with control. Study revealed that, in spite of the molecular parameters being affected at varied level, overall ovarian physiology is maximally affected in IR and Cd coexposed group, thus mimicking the condition similar to those prevailing in infertile females.

## 1. Introduction

Development and function of female reproductive tract depend on coordinated biological processes. Follicular development and ovulation are dependent on proliferation and differentiation of both granulosa and theca cells that undergo steroidogenesis upon stimulation with gonadotropins and intraovarian cytokines. Granulosa cells of the ovary are very important components that are involved in the production of sex steroid hormones and a milieu of growth factors involved in interaction with oocyte development. Cellular growth in ovary is controlled by apoptosis of granulosa cells, a phenomenon responsible for follicular atresia. Interference with any of these processes can predispose women to reproductive dysfunctions ultimately leading to infertility [1]. It has been estimated that approximately 15% of the population in industrially developed countries are infertile [2]. Among the various proposed etiological factors for infertility, genetic, immunologic, endocrinology, and environmental disorders account for 50% of the patients; however, in the remaining 50%, the cause remains unknown (idiopathic infertility) [3, 4]. It is now thought that the idiopathic infertility might be due to lifestyle-environment interaction.

Changes in lifestyle with high carbohydrate diet intake or sedentary habits lead to insulin resistant (IR) condition. It has been reported that approximately 50–75% of women with polycystic ovarian syndrome (PCOS) have some degree of IR. Furthermore, it has been found that women with IR are more likely to have PCOS [5]. Besides the classical target organs for insulin action such as liver, adipose tissue, and muscle, the presence of insulin receptors in both stromal and follicular compartments and findings for insulin's ability to stimulate steroidogenesis in ovarian cells* in vitro* have eventually established ovary as an important target organ for insulin action [6]. Hyperinsulinemia in IR alters the genes, enzymes, and proteins that are crucial for the steroidogenic machinery ultimately affecting follicle development and ovulation [7].

Amongst many environmental pollutants, cadmium (Cd) is an endocrine disruptor which is relatively dispersed in the environment mainly because of pollution from a variety of sources, including mining, smelting, fossil fuel combustion, batteries, paints, plastics, and tobacco smoke [8]. Because of high rate of soil-to-plant transfer, Cd is a contaminant found in most human foodstuffs, which renders diet a primary source of exposure among nonsmoking, nonoccupationally exposed populations [9]. Due to its unknown biological function, low rate of excretion from the body, and long biological half-life, it accumulates over time in blood, kidney, liver, and the reproductive organs such as placenta, testis, and ovaries [10]. Using the benchmark dose- (BMD-) derived urinary Cd threshold, the tolerable weekly intake for Cd was 2.5 *μ*g/Kg body weight, which corresponds to 25 *μ*g/day for a person who weighs 70 kg [11]. The stimulatory or inhibitory effects of Cd on key ovarian steroidogenic enzymes at a subclinical dose of 0.05 mg/kg body wt. are very well established in ovary and granulosa cells in our lab [12–15]. In recent times, our lab has also demonstrated decrease in expression of StAR in granulosa cells of F1 generation rats that were exposed to Cd during their developmental age [16]. Decrease in gene expression of CYP19A1 due to Cd exposure has been established in carp ovarian follicles [17]. Apart from reproductive disorder, results of both human and animal studies suggest an association between Cd exposure, elevated blood glucose levels, and the development of diabetes [18].

To date, studies have mainly focused on investigating the effect of Cd and IR individually on granulosa cells ultimately affecting fertility. It has been observed that Cd and IR are prevalent in today's lifestyle. In light of this, we aim to investigate the effect of Cd on dexamethasone, a glucocorticoid, induced IR granulosa cells, which could be mimicking present environment condition and sedentary life on the reproductive physiology at both cellular and molecular levels. There is ample evidence, in numerous species, that glucocorticoids result in increased serum insulin and glucose, decreased insulin-mediated glucose uptake, and insulin resistance [5, 19, 20]. We further evaluated certain physiological parameters along with mRNA and protein expression of StAR for cholesterol transport and CYP19A1 and 17*β*-HSD responsible for steroid synthesis in granulosa cells of IR animals exposed to Cd.

## 2. Materials and Methods

### 2.1. Hormones and Reagents

Pregnant mare serum gonadotrophin hormone (PMSG) and trypan blue were purchased from Sigma Chemical Co. (USA). All the other chemicals used were of analytical grade and were purchased from Sisco Research Laboratories Ltd. (Mumbai, India). Glucose estimation kit glucose oxidase-peroxidase (GOD-POD) was from Reckon Diagnostics. Rat Insulin ELISA kit and serum estradiol kit were procured from Mercodia and Abbott, respectively.

### 2.2. Antibodies

Rabbit polyclonal antibody against CYP19A1 and *β*-actin was purchased from cell signaling. Rabbit polyclonal antibodies against 17*β*-HSD and StAR protein were generous gift from Dr. Vann Luu-The (CHUL Research Center and Laval University, Canada) and Dr. Douglas M. Stocco (Department of Cell Biology and Biochemistry, Texas Tech University, Lubbock, Texas, USA), respectively.

### 2.3. Animals and Experimental Design

**Figure 1:**
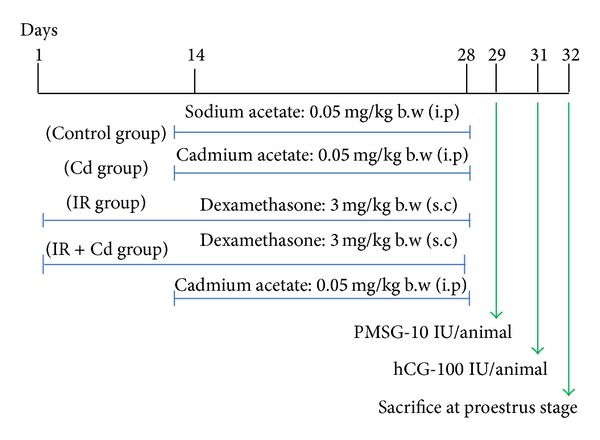
Time line for sodium acetate, Cd, dexamethasone, PMSG, and hCG injections in rats.

All rats used in these studies were of the* Charles Foster* strain and were bred in our own animal facility. Animals were maintained under standard laboratory conditions (temperature: 24 ± 2°C; light: 12-h light/12-h dark) and were given standard pellet diet and water ad libitum throughout the experimental period. Adult virgin female* Charles Foster *rats weighing 180–220 g were used for this study and were monitored regularly for each stage of estrous cycle. Animals were divided into four groups with 6 animals in each group and the doses were given as shown in Figure 1. The dosage was selected on the basis of previous reports [12, 19]. After 28 days of dexamethasone treatment, animals showing IR were chosen for the study and sacrificed at proestrus stage after superovulation induction with PMSG-10 IU and human chorionic gonadotrophin (hCG-100 IU) hormones. The experimental study was approved by the Animal Ethical Committee of the Department of Biochemistry, the M.S. University of Baroda, and was in accordance with the CPCSEA norms.

### 2.4. Selection of IR Animals and Confirmation of IR

Standard oral glucose tolerance test was performed after 28 days of dexamethasone injection. After 12-hour fasting, 300 *μ*L of blood was collected from retro-orbital sinus for glucose and immunoreactive insulin measurement followed by oral administration of 2 gm/kg body weight glucose and 100 *μ*L blood collection at 30, 60, 90, and 120 min for OGTT. The blood was subjected to 4000 rpm for 10 min and serum was separated. Glucose was estimated from serum using GOD-POD kit as per the manufacturers instructions. Fasting serum was then proceeded for determining insulin levels using Rat Insulin ELISA kit according to manufacturers protocol (Mercodia, Germany). The fasting insulin resistance index (FIRI), a measure of the insulin sensitivity, was calculated according to the following formula:

 FIRI = fasting serum insulin (*μ*IU/mL) × fasting serum glucose (mmol/L)/25.

FIRI has been shown to have a better correlation with insulin sensitivity values than the fasting glucose/insulin ratio giving a reference index value as shown in Table 1 [21].

### 2.5. Estrous Cyclicity

Estrous cyclicity was monitored throughout the dosage schedule. Vaginal lavages from female rats were obtained and viewed under a microscope daily (between 0900 and 1000 h) for at least three consecutive cycles. A normal estrous cycle was defined as exhibiting vaginal cytology that was leukocytic (diestrus) for 2 days followed by nucleated cells (proestrus) for 1 day, cornified cells (estrus) for 1 day, and mixed cells (metestrus) for 1 day.

### 2.6. Serum Estradiol

Estradiol concentrations were measured from rat serum by commercially available kit (Architect Estradiol, Abbott, Ireland) using chemiluminescent microparticle immunoassay technology. The resulting chemiluminescent reaction was measured as relative light units (RLUs) that were detected by the ARCHITECT *i* optical system.

### 2.7. Histological Analysis

One ovary from each group was removed and stored in 10% formaldehyde and then proceeded for sectioning and hematoxylin and eosin staining. Histological observations were made microscopically. The slides were observed for mature follicle, fibrosis, and other morphological changes.

### 2.8. Granulosa Cell Isolation

Granulosa cells were isolated at proestrus stage from the rat ovary as explained earlier [22]. Briefly, ovaries were removed from animals and kept in Hanks balanced salt solution (HBSS) and centrifuged at 1000 rpm and 4°C to remove all the fat. The ovaries were then incubated in EGTA-BSA solution for 15 min at 37°C followed by centrifugation at 1000 rpm for 5 min. Samples were then incubated in hypertonic sucrose solution for 5 min at 4°C and then centrifuged at 1500 rpm. The granulosa cells were expressed from ovary in HBSS by blunt spatula and then washed three times with HBSS-EGTA by centrifugation at 1500 rpm for 5 min. The viability of cells was analysed at final stage by trypan blue exclusion dye method.

### 2.9. Total RNA Extraction and RT-PCR

Total RNA was isolated from granulosa cells by using TRIzol (Sigma-Aldrich, USA). Purity of RNA was confirmed by *A*260/280 ratio and checked for integrity. 2 *μ*g of total RNA was reverse transcribed into first strand cDNA and subjected to PCR amplification for various genes as mentioned in Table 2 [23]. Gradient PCR was performed with a range of annealing temperature from 51 to 60°C. cDNA was amplified for 35 cycles using Fermentas 2x master mix (1.5 unit Taq polymerase, 2 mM dNTP, 10x Tris, glycerol reaction buffer, 25 mM MgCl_2_) with 20 pM forward and reverse primer. *β*-Actin served as internal control and negative RT was performed with untranscribed RNA. PCR products were separated on 15% polyacrylamide gels (Sigma-Aldrich, USA) and visualized and images were captured with Alpha Imager software (UVP Image Analysis Software Systems, USA) for densitometric analysis.

### 2.10. Western Blot Analysis

Granulosa cells were suspended in 62.5 mM Tris-HCl, pH 6.8, 6 M urea, 10% (v/v) glycerol, 2% (w/v) SDS, 0.00125% (w/v) bromophenol blue, and freshly added 5% (v/v) *β*-mercaptoethanol and subjected to sonication on ice. Total protein content was quantified using Bradford assay (Bio-Rad Bradford Solution, USA). 20 *μ*g protein was loaded on 10% SDS-polyacrylamide gel electrophoresis under reducing conditions, along with prestained molecular weight markers. The separated proteins were electrophoretically transferred onto a nitrocellulose membrane (GE Healthcare) by a wet method (Bio-Rad, USA). The transfer was performed at a constant voltage (100 V) for 90 min in a buffer consisting of 25 mM Tris, 192 mM glycine, and 20% methanol. The membrane was then incubated for 1 h at room temperature in blocking buffer (TBS-containing 5% skimmed milk and 0.1% Tween-20). Then, the membranes were incubated overnight at 4°C with appropriate antibody at a dilution of 1 : 1000 in TBS-containing 5% skimmed milk and 0.1% Tween-20. They were washed in PBS-0.1% Tween-20 and incubated for 1 h at room temperature with a horseradish peroxidase-conjugated anti-rabbit or anti-mouse IgG (final dilution 1 : 5000; Bangalore Genei) in TBS-containing 5% skimmed milk and 0.1% Tween-20. The signal was detected by ECL (enhanced chemiluminescence, Millipore Inc., USA).

### 2.11. Statistical Analysis

The results are presented as mean ± standard error mean. The data were statistically analyzed by employing one-way analysis of variance followed by Bonferroni's multiple comparison test (GraphPad Prism; Graph Pad Software, Inc., La Jolla, CA). The minimum level of significance (*P* < 0.05) was considered.

## 3. Results

### 3.1. Induction of IR Is Solely because of Dexamethasone

**Figure 2:**
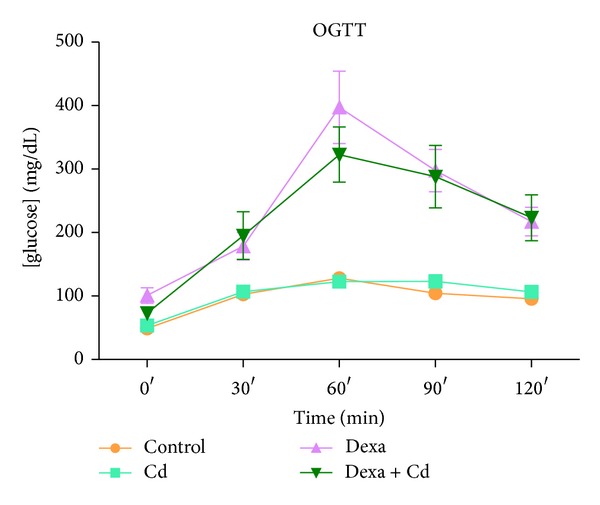
Effect of Cd, dexamethasone, and dexamethasone + Cd treatment on the OGTT profile of rats. Serum glucose levels were measured using GOD-POD. Data presented as mean ± SEM of three independent observations (*n* = 6). Dexa = dexamethasone.

**Figure 3:**
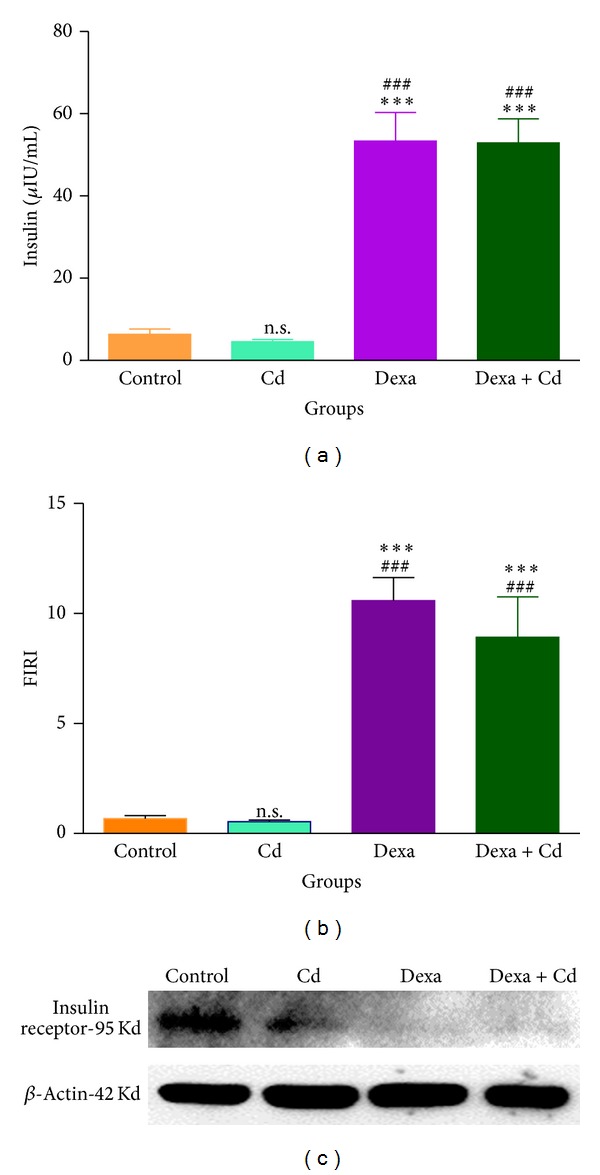
Effect of dexamethasone and Cd either alone or in combination on insulin resistance in terms of (a) serum insulin levels, (b) FIRI, and (c) protein expression of insulin receptor in granulosa cell lysate using beta actin as internal control in rats. The values are represented as mean ± SEM of three independent observations (*n* = 6). Serum insulin levels and FIRI were significantly increased (***P < 0.001 and ^###^ *P* < 0.001) in Dexa group as compared to control and Cd group. Dexa + Cd also showed significant increase (***P < 0.001 and  ^###^ *P* < 0.001) in serum insulin and FIRI as compared to control and Cd group. Dexa = dexamethasone.

The groups treated with dexamethasone alone and in combination with Cd showed significant glucose intolerance as shown in OGTT curve compared to control, whereas Cd alone did not show any change as compared with control (Figure 2). Fasting serum insulin levels and FIRI values were observed to be significantly high (*P* < 0.001) in groups treated with dexamethasone alone and in combination with Cd with respect to control, whereas Cd alone did not show any change (Figures 3(a) and 3(b)). Further, to confirm the status of IR at granulosa cell level, western blot for insulin receptor was performed. We observed significant downregulation of insulin receptor in granulosa cells of dexamethasone alone and coexposed group as compared to control (Figure 3(c)).

### 3.2. Effect of Cd and IR on Serum Estradiol Concentrations

**Figure 4:**
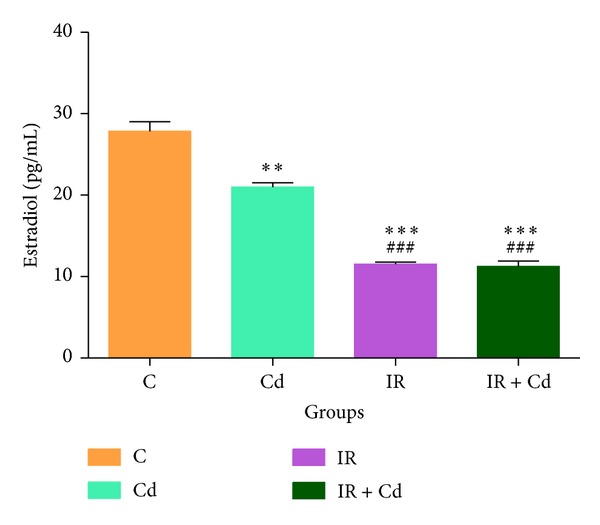
Effect of IR and Cd either alone or in combination on serum estradiol levels. The values are represented as mean ± SEM of three independent experiments (*n* = 6). Estradiol was observed to be significantly decreased in Cd, IR, and IR + Cd group (**P < 0.01, ***P < 0.001, and ***P < 0.001) as compared to control group. IR and IR + Cd group showed significant decrease in estradiol (^###^ *P* < 0.001 and ^###^ *P* < 0.001) as compared to Cd group.

Estradiol concentration was observed to be significantly decreased in Cd (*P* < 0.01), IR (*P* < 0.001), and IR + Cd (*P* < 0.001) group as compared to control group. When compared between the groups, both IR and IR + Cd demonstrated significant decrease compared to Cd (*P* < 0.01) treated group (Figure 4).

### 3.3. Effect of Cd and IR on Estrous Cyclicity

**Figure 5:**
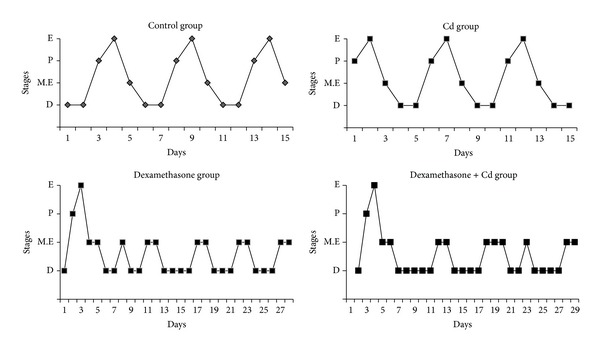
Effect of dexamethasone and Cd either alone or in combination on estrous cyclicity of rats (*n* = 6). E = estrous stage-cornified cells, P = proestrous stage-nucleated epithelial cells, M.E = metestrus-nucleated, cornified and leucocytes, and D = diestrous stage-leucocytes.

Daily inspection of vaginal cytology in the groups treated with dexamethasone and its coexposure with Cd revealed absence of normal estrous cyclicity, with a prolonged period of persistent mixed cells (metestrus), which last for few days, followed by a period of persistent leucocytes (diestrus) as compared with control group. However, Cd group alone did not show any change in estrous cyclicity (Figure 5).

### 3.4. Histological Analysis Revealed Alterations in Ovary

**Figure 6:**
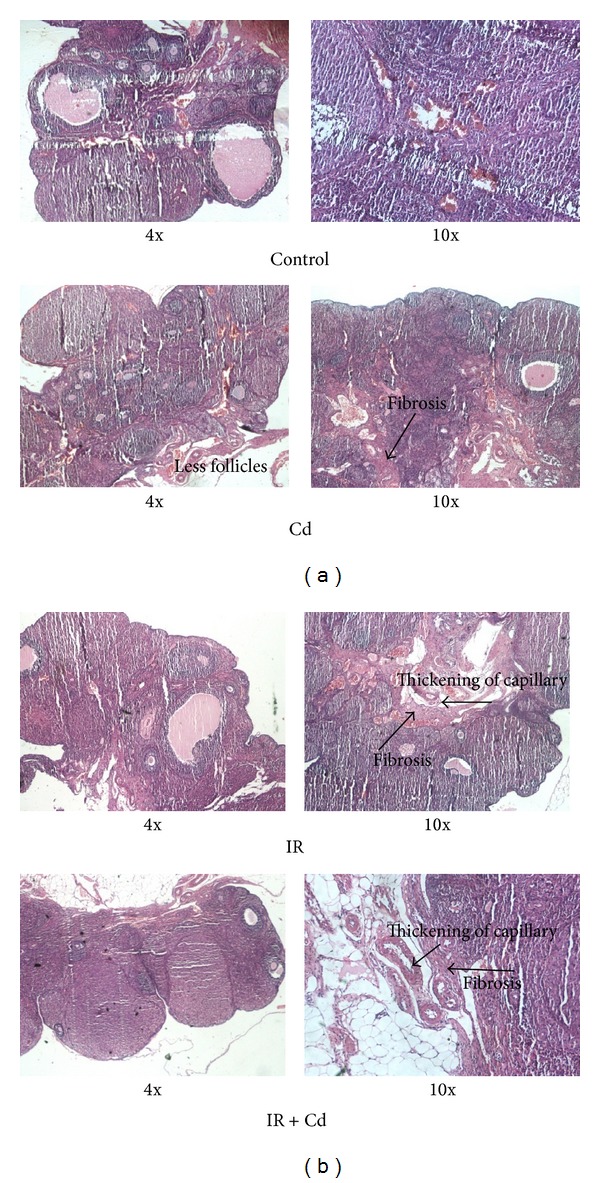
Histological assessment of ovarian structure in control, Cd, IR, and IR + Cd treated rats. (*n* = 4). Ovarian sections were stained with hematoxylin and eosin. A decrease in mature follicle in IR and IR + Cd group is compared with control group. Fibrosis (arrow) is detected in Cd, IR, and IR + Cd group when compared with control group. In IR and IR + Cd group increase in capillary thickness (arrow) can be detected.

Histological examination of ovaries showed absence of mature follicles in all the three groups as compared to control group. Increased amount of fibrosis in the stroma was also observed in all the three groups as compared to control. Thickening of capillary wall was found to be evident in IR and IR + Cd treated group as compared to control group (Figure 6).

### 3.5. Granulosa Cell Viability Assay

**Figure 7:**
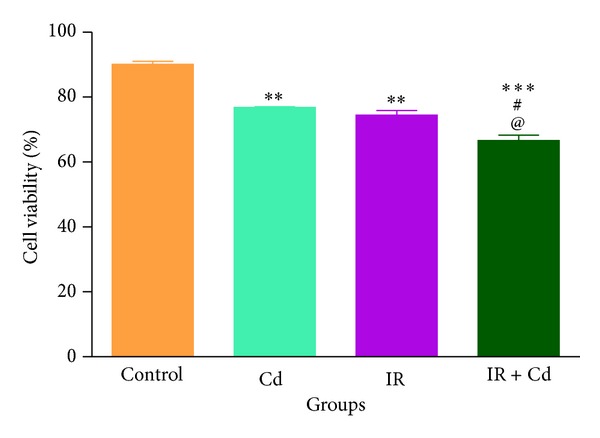
Effect of IR and Cd on % cell viability of luteinized granulosa cells of rats. The values are represented as mean ± SEM of three independent experiments (*n* = 6). Granulosa cells showed significant decrease in % viability (**P < 0.01, **P < 0.01, and ***P < 0.001) when compared to control. IR + Cd group further showed significant decrease in % granulosa cell viability (^#^ *P* < 0.05 and ^@^ *P* < 0.05) when compared to Cd and IR.

Further investigation of granulosa cell viability by trypan blue exclusion dye demonstrated deleterious effect of Cd and IR either alone or in combination. Percentage cell viability was significantly affected in all the groups as compared to control groups. The Cd treated group in combination with IR exhibited maximum decrease (*P* < 0.001) whereas Cd and IR groups exhibited significant decrease (*P* < 0.01) in granulosa cell viability as compared to control group. Within the groups, the combined group showed significant decrease (*P* < 0.05) as compared to Cd alone and IR group and no change was observed between IR and Cd alone group (Figure 7).

### 3.6. StAR, CYP19 A1, and 17*β*-HSD Expression in Rat Granulosa Cells

**Figure 8:**
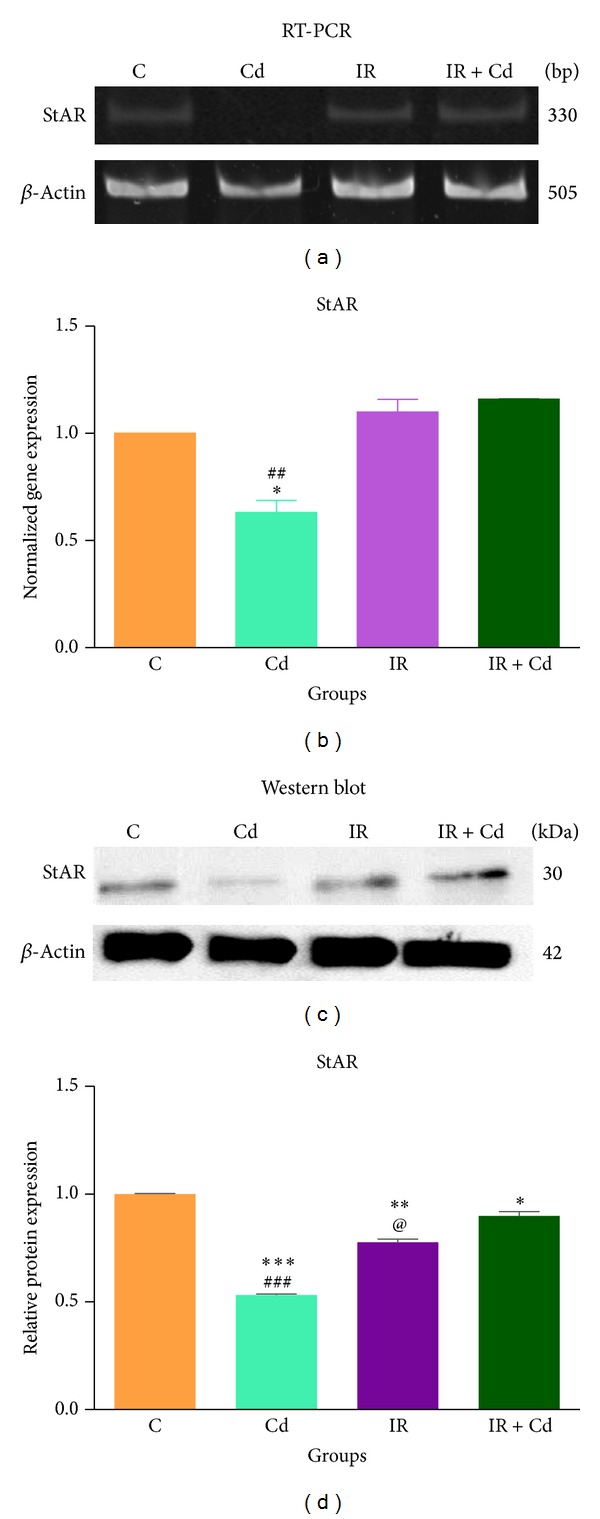
Effect of IR and Cd either alone or in combination on StAR (a) mRNA expression, (b) densitometric analysis for mRNA, (c) protein expression, and (d) densitometric analysis for protein. The normalized expression values are represented as mean ± SEM of three independent experiments (*n* = 6). Cd demonstrated significant decrease in StAR mRNA expression (*P < 0.05 and ^##^ *P* < 0.01) when compared to control, IR, and IR + Cd group. StAR protein expression was significantly decreased (***P < 0.001, **P < 0.01, and *P < 0.05) in Cd, IR, and IR + Cd group when compared to control, (^###^ *P* < 0.001) in Cd group when compared to IR group, and (^@^ *P* < 0.05) in IR group when compared to IR + Cd group.

**Figure 9:**
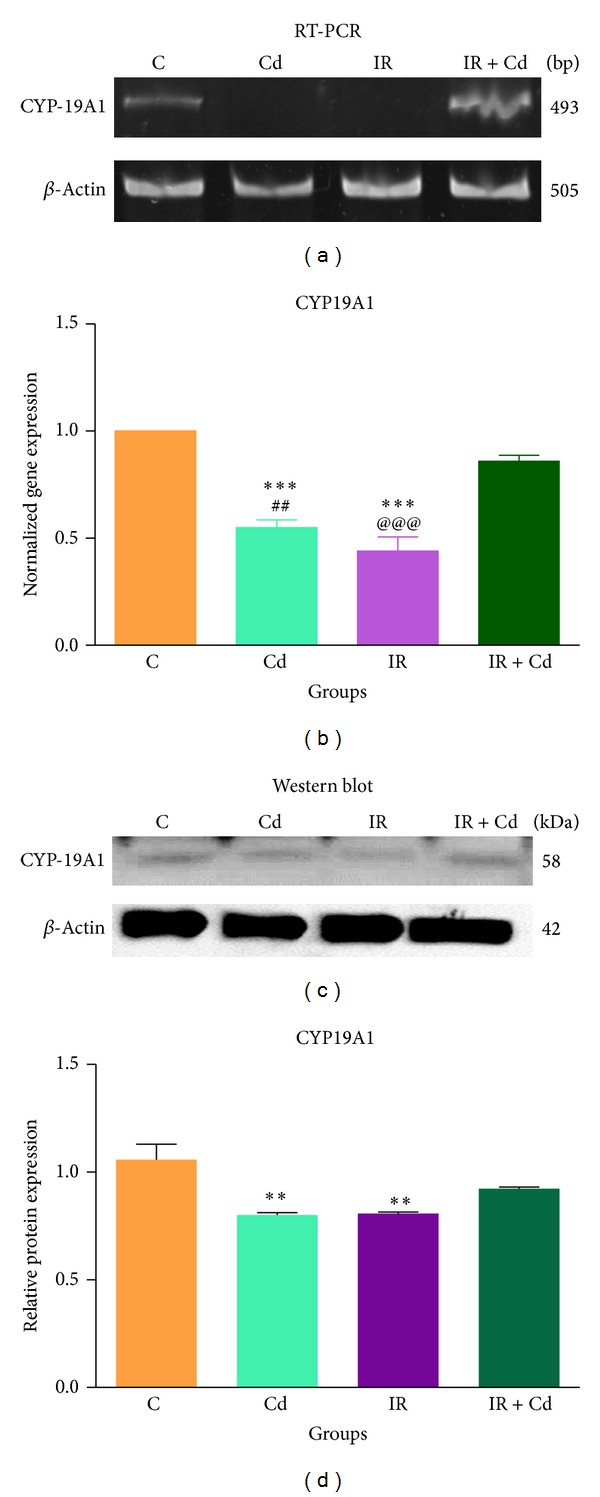
Effect of IR and Cd either alone or in combination on CYP19A1 (a) mRNA expression, (b) densitometric analysis for mRNA, (c) protein expression, and (d) densitometric analysis for protein. The normalized gene expression values are represented as mean ± SEM of three independent experiments (*n* = 6). Cd and IR groups showed significant decrease (***P < 0.001) in CYP19A1 mRNA expression as compared to control. Cd and IR showed significant decrease (^##^ *P* < 0.01 and ^@@@^ *P* < 0.001), respectively, in CYP19A1 mRNA expression when compared to IR + Cd. CYP19A1 protein was significantly decreased in Cd and IR group (**P < 0.01) as compared to control.

**Figure 10:**
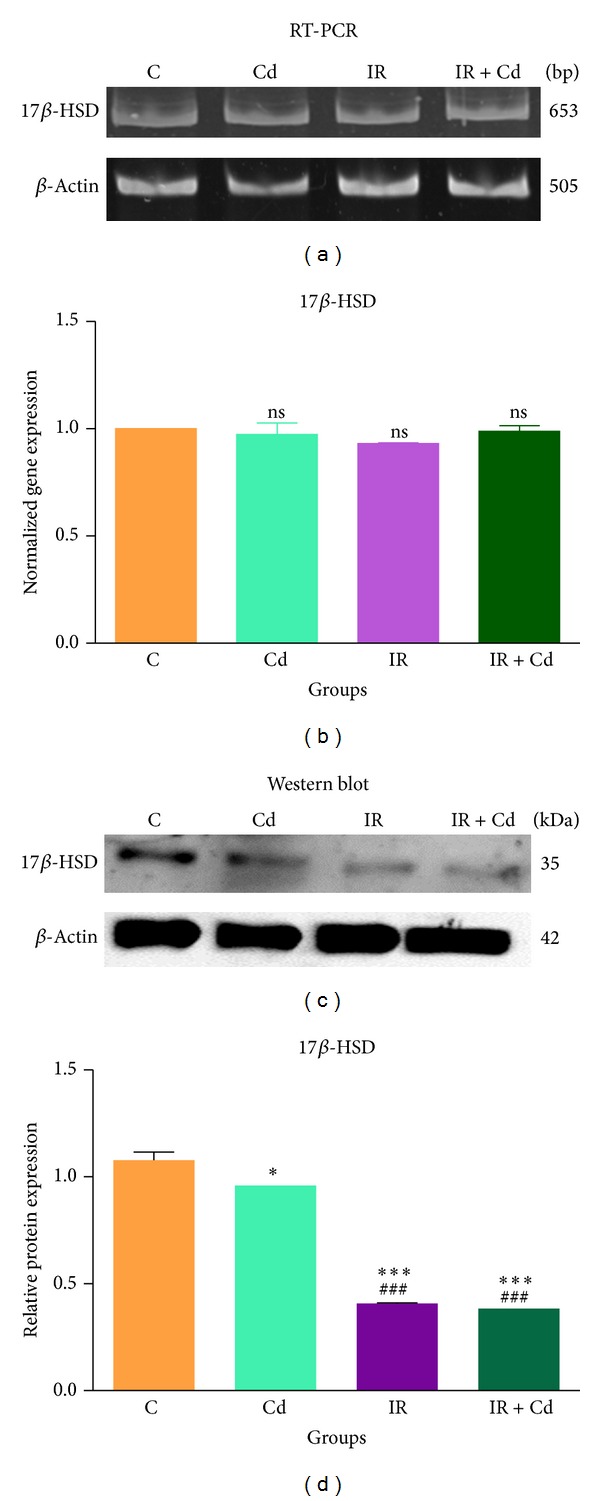
Effect of IR and Cd either alone or in combination on 17*β*-HSD (a) mRNA expression, (b) densitometric analysis for mRNA, (c) protein expression, and (d) densitometric analysis for protein (*n* = 6). The normalized gene expression values are represented as mean ± SEM of three independent experiments. ns = not significant. Cd showed significant decrease (*P < 0.05) in 17*β*-HSD protein expression as compared to control. IR and IR + Cd groups showed significant decrease (***P < 0.001 and ^###^ *P* < 0.001) in 17*β*-HSD protein expression as compared to control and IR.

To assess the effects of Cd, IR, and their coexposure on the granulosa cell steroidogenic transcriptional machinery, mRNA and protein expression of StAR, CYP19A1, and 17*β*-HSD were analyzed by RT-PCR and western blot employing *β*-actin as the internal control. As expected mRNA and protein expression of StAR showed significant decrease (*P* < 0.05 and *P* < 0.001), respectively, in Cd treated group as compared to control (Figures 8(a) and 8(b)). There was slight but significant decrease (*P* < 0.05) in protein expression of StAR in IR + Cd group whereas animals exposed to IR alone demonstrated an intermediate pattern of inhibition (*P* < 0.01) as compared with control (Figures 8(c) and 8(d)). As shown in Figures 9(a) and 9(b), significant decrease (*P* < 0.001 and *P* < 0.01) was observed in mRNA expression of CYP19A1 in Cd alone when compared to control and IR + Cd group, respectively. Similarly IR group showed significant decrease (*P* < 0.001) in mRNA expression of CYP19A1 as compared to control and IR + Cd animals. IR + Cd group showed nonsignificant decrease in CYP19A1 mRNA as compared to control group. CYP19 A1 protein revealed significant decrease (*P* < 0.01) in Cd alone and IR groups, respectively, as compared to control. CYP19A1 protein showed nonsignificant decrease in IR + Cd group as compared to control (Figures 9(c) and 9(d)). Further, mRNA expression of 17*β*-HSD did not reveal any significant difference in any of the groups (Figures 10(a) and 10(b)). But protein expression of 17*β*-HSD revealed significant decrease (*P* < 0.05) in Cd group and maximum decrease (*P* < 0.001) in IR and IR + Cd group as compared to control (Figures 10(c) and 10(d)), whereas IR and IR + Cd revealed significant decrease (*P* < 0.001) as compared to Cd group with no change between themselves.

## 4. Discussion

Evidences state that, because of the change in lifestyle, there are increasing number of women with some degree of IR [5]. Moreover, due to environmental issues, women are also exposed to toxicants such as Cd in their reproductive age [9, 24]. As evident in present scenario, females are eventually exposed to both Cd and IR condition which may lead to altered reproductive performance and fertility-related problems. In this regard, the present study analyzed the extent of the effect of Cd on the ovary of dexamethasone induced IR rat model.

To prove this, at a very first step, we developed an IR female rat model by using dexamethasone. Dexamethasone has been previously reported to exacerbate IR in monovular animal model, such as cow, in isolated and cultured 3T3 adipocytes and in ovarian theca cells from porcine follicles [5, 20]. Further, studies on Cd exposure on animal models have shown Cd as a diabetogenic agent when injected at 2.0 mg/kg daily for 14 days [18]. The results of the present investigation demonstrated glucose intolerance and IR in the groups treated with dexamethasone alone and in combination with Cd. In our experimental observations, we found that Cd treated group did not show any glucose intolerance and insulin resistance indicating that the subclinical dose of 0.05 mg/kg b.w for 15 days used in the present study might not be sufficient to exacerbate type II diabetes. These findings thus indicate that development of IR in coexposed group is because of dexamethasone. Further, examination of IR status with western blot analysis in granulosa cell revealed downregulation of insulin receptor in groups treated with dexamethasone and dexamethasone with Cd as compared to control and Cd alone treated animals. In earlier reports, insulin receptor has been demonstrated in cultured porcine granulosa and theca cells by monitoring protein expression of downstream targets such as IRS-1, GLUT-4, and PPAR-*γ* [20, 25]. We instead demonstrated downregulation of insulin receptor itself in dexamethasone treated granulosa cells confirming the establishment of frank IR condition in rodent model at the cellular level itself.

Fertile rats show normal estrous cyclicity of 4 days and a normal ovarian morphology with growing follicles and a well-defined stroma [26]. Prolonged metestrus and diestrus stages were observed in IR and IR + Cd group which correlated with less number of mature follicles indicating direct or indirect actions of IR on the follicles. Recently glucocorticoid induced insulin resistance has been shown to suppress circulating estradiol concentration in serum [5]. Our present result of Cd treated group is similar to earlier reports from our lab which demonstrated reduced serum estradiol and normal estrous cyclicity with subclinical Cd exposure [16]. However, insulin resistance condition in IR and IR + Cd group can be correlated with abnormal cyclicity, decreased serum estradiol concentration, and very less number of mature follicles which is responsible for anovulation and reduced reproductive capacity and ultimately leading to reproductive failure. These results are substantiated with earlier reports where IR as such has been shown to have an effect on oocyte development, ovulation, fertilization, and implantation thus leading to infertility [5, 27]. These effects of the coexposure of Cd and IR further inquisited us to investigate their effect at cellular and molecular levels.

Folliculogenesis and ovulation being the ultimate functions of the ovary are intimately linked to multidirectional communication between oocyte, granulosa cells, and theca cells [28]. Granulosa cells are the first somatic cell type known to interact with germ cells [29] and are involved in apoptosis and steroidogenesis [30]. Previous reports have demonstrated decrease in granulosa cell number and change in its morphology with* in vitro* cadmium exposure [14, 31, 32]. Actions of insulin on granulosa cells have also been implicated in PCOS, where granulosa cell numbers were found to be decreased relative to follicle size [33]. Our data for reduced cell viability along with the altered histological findings imply that Cd exposure along with IR alters folliculogenesis and granulosa cell viability more deleteriously in coexposed group.

The rate-limiting step in ovarian steroid hormone synthesis requires StAR protein in order to transport cholesterol to the intramitochondrial membrane [34]. Cd alone demonstrated decreased expression of StAR at both mRNA and protein level when compared to control group supporting the fact that StAR might be a potent target for Cd to bind and thus alter steroidogenesis [10, 16, 35, 36]. IR alone failed to demonstrate any effect on expression of StAR mRNA but showed a significant decrease in its protein expression [37]. Similarly little but significant decrease in protein expression of StAR in IR + Cd group in the present study indicated reduced gonadal steroidogenesis leading to accumulation of cholesterol in lipid droplets supported by earlier reports [38].

In follicular steroidogenesis, the synthesis of estrogen is dependent on CYP19A1 and 17*β* HSD whose expressions are highly dependent on the type of granulosa cells [39]. In our study, we demonstrated significant decrease in protein expression of 17*β* HSD and a nonsignificant decrease in protein expression of CYP19A1 in the IR + Cd group as compared with control group. These changes along with attenuation of estradiol hormone synthesis disrupt the normal developmental program thereby arresting estrus cyclicity in metestrus and diestrus stage. The signaling pathway in granulosa cells from IR ovaries gets altered forcing the cell to luteinize at a premature stage as compared to granulosa cells from control ovaries. This leads to terminal differentiation in these cells because of which they lose their proliferative capacity and thus fail to express CYP19A1 and 17*β* HSD [5, 6]. Also presence of proteinaceous substances such as TGF-*β*, IGF-1, EGF, TNF-*α*, abnormally high levels of 5 alpha-androstane-3, 17-dione in the follicular fluid of insulin resistant patients act as endogenous inhibitors of estrogen production by inhibiting CYP19A1 activity explaining the potential for decrease in estradiol synthesis independent of CYP19A1 protein expression in IR + Cd group. Studies in the literature have also demonstrated strong inhibition of CYP19A1 expression and activity by Cd and other endocrine disruptors in many different species such as Teleost Fish and human embryonic 293 cells thus leading to decreased estradiol synthesis and disturbing reproductive cycle [17, 37, 40, 41].

**Figure 11:**
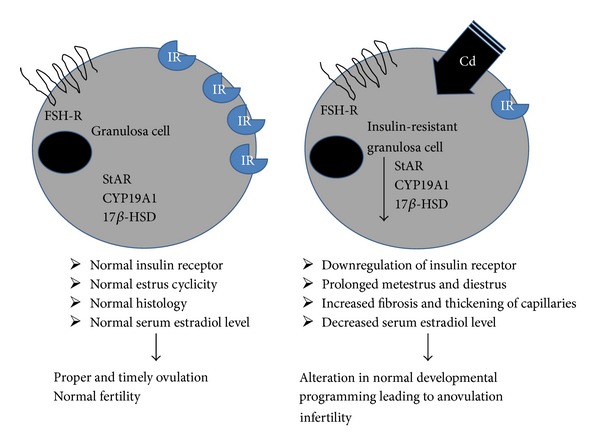
Schematic diagram summarizing potential effect of Cd on dexamethasone induced IR in granulosa cells of rat.

Summarizing the results as per the insults, Cd alone directly interacts with StAR ultimately leading to a decrease in availability of cholesterol. It also decreases 17*β* HSD and CYP19A1 protein expression thus leading to relative decrease in estradiol production along with fibrosis. IR alone decreases expression of StAR, 17*β* HSD, and CYP19A1 protein leading to decrease in estradiol levels along with fibrosis and thickening of capillaries; the effects seem to be more adverse as compared to Cd alone. In IR + Cd, along with decrease in protein expression of StAR, 17*β* HSD, and CYP19A1, there is significant decrease in granulosa cell viability as compared to Cd alone and IR groups, ultimately decreasing estradiol levels (Table 3). This shows that although individual parameters were variedly affected at molecular level in different groups, overall effect is more deleterious in coexposed group providing support for their involvement in etiology of reproductive dysfunction as shown in schematic Figure 11.

## 5. Conclusion

This pilot study demonstrates that ovarian morphology and normal steroidogenic program in IR + Cd group is altered, causing destruction of the normal developmental programing in ovary. This research seeks attention towards understanding the insulin resistance along with endocrine disruptors. Thus the presence of various environmental pollutants with change in life style is alarming signals for increased incidence of female infertility which might have imprinting effect on the next generation.

## Figures and Tables

**Table 1:** Reference value for FIRI.

Index value	Indication
Less than 2.67	Normal
Between 2.93 and 3.12	Typically found in persons who are obese and may indicate insulin resistance
Above 3.22	Indicates prediabetes or type-II diabetes

**Table 2:** Details of genes, accession number, primers, annealing Tm, and expected size of the PCR-amplified c-DNA.

Gene name	GenBank accession number	Sequence of the primer Fw primer Rv primer	Annealing temperature (°C)	Product size (bp)
StAR	NM_ 31558	5′ AGGCAGGGGGATCTTTCTAA 3′ 5′ TGCCTGACTAGGGTTTCGTT 3′	56.8	330
17*β*-HSD	AF035156	5′ CCTCCTTCGCCACTATCAGC 3′ 5′ GGAGACAAATGAGGGCTC 3′	55.4	653
CYP19A1	NM_017085	5′ GGAATCCATCAAGCAGCATT 3′ 5′ TTCCACCTCCGGATACTCTG 3′	58	493
*β*-Actin	V01217	5′ CCTGCTTGCTGATCCACA 3′ 5′ CTGACCGAGCGTGGCTAC 3′	57	505

**Table 3:** Summary of the results.

Parameters	Groups		
	Cd	IR	IR + Cd
IR status	—	↓↓↓	↓↓↓
Serum estradiol	↓	↓↓	↓↓
Histology	Fibrosis	Fibrosis and thickening	Fibrosis and thickening
Granulosa cell viability	↓	↓	↓↓
StAR-mRNA	↓↓↓	—	—
StAR-protein	↓↓↓	↓↓	↓↓
CYP19 A1-mRNA	↓↓	↓↓	↓ (ns)
CYP19 A1-protein	↓↓	↓↓	↓ (ns)
17-*β* HSD-mRNA	—	—	—
17-*β* HSD-protein	↓	↓↓	↓↓

(— represents no change, down arrow represents downregulation, and ns represents nonsignificant).
